# Room Temperature Relaxometry of Single Nitrogen Vacancy Centers in Proximity to α-RuCl_3_ Nanoflakes

**DOI:** 10.1021/acs.nanolett.3c05090

**Published:** 2024-04-08

**Authors:** Jitender Kumar, Dan Yudilevich, Ariel Smooha, Inbar Zohar, Arnab K. Pariari, Rainer Stöhr, Andrej Denisenko, Markus Hücker, Amit Finkler

**Affiliations:** † Department of Chemical and Biological Physics, Weizmann Institute of Science, 7610001 Rehovot, Israel; ‡ Department of Condensed Matter Physics, Weizmann Institute of Science, 7610001 Rehovot, Israel; § 3rd Institute of Physics, IQST and ZAQuant, University of Stuttgart, 70569 Stuttgart, Germany

**Keywords:** NV center, quantum sensing, *T*_1_ relaxometry, quantum spin liquid, 2D materials

## Abstract

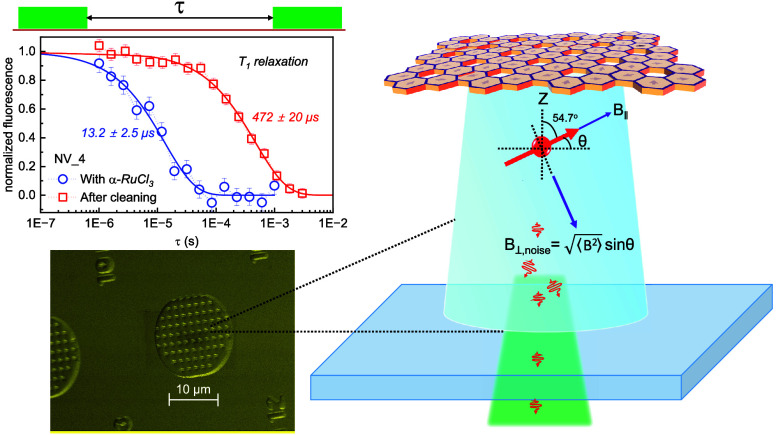

Nitrogen vacancy (NV) center-based magnetometry has been proven to be a versatile sensor for various classes of magnetic materials in broad temperature and frequency ranges. Here, we use the longitudinal relaxation time *T*_1_ of single NV centers to investigate the spin dynamics of nanometer-thin flakes of α-RuCl_3_ at room temperature. We observe a significant reduction in the *T*_1_ in the presence of α-RuCl_3_ in the proximity of NVs, which we attribute to paramagnetic spin noise confined in the 2D hexagonal planes. Furthermore, the *T*_1_ time exhibits a monotonic increase with an applied magnetic field. We associate this trend with the alteration of the spin and charge noise in α-RuCl_3_ under an external magnetic field. These findings suggest that the influence of the spin dynamics of α-RuCl_3_ on the *T*_1_ of the NV center can be used to gain information about the material itself and the technique to be used on other 2D materials.

The NV center is a defect in the diamond lattice that forms an electronic qubit with a sufficiently long coherence time for a variety of applications, ranging from quantum computing and quantum information to quantum sensing.^1−5^ In the realm of sensing, the NV center serves as a highly sensitive sensor for many physical quantities, including temperature, rotation, electric and magnetic fields, and pressure.^6,7^ In magnetic field sensing, the NV center is susceptible not only to AC and DC fields but also to magnetic fluctuations across a broad frequency spectrum.^8^ For instance, the transverse coherence times *T*_2_^*^ and *T*_2_ of the NV center are susceptible to DC to MHz magnetic noise, depending on the pulse sequences used, such as Ramsey, Hahn echo, and CPMG.^9^ Complementing this, noise generated from fluctuations in the GHz regime affects the longitudinal relaxation time *T*_1_ of the NV center.^10^

Magnetic noise sensing finds useful applications in the study of 2D materials. Indeed, intense research has been directed toward van der Waals (vdW) strongly correlated materials in condensed matter physics. A weak interlayer vdW coupling and strong anisotropic magnetic interactions allow 2D materials to host interesting quantum properties in exfoliated single to few atomic layers such as layer-dependent magnetism (ferro/anti), multiferroicity, superconductivity, and quantum spin liquid state.^11−14^ Conventional experimental techniques widely used to explore low-dimensional magnetic systems include spin-polarized scanning tunneling microscopy,^15^ scanning SQUID magnetometry,^16^ X-ray magnetic circular dichroism,^17^ and neutron scattering.^18^ Common to these techniques are the requirements of low temperatures and limited detection bandwidth for magnetic fluctuations. Moreover, in some, for instance, the routinely used magnetic probe neutron scattering technique, a relatively large amount of sample is required, and it is also susceptible to high absorption in heavy elements, such as iridium. In recent studies on 2D magnetic materials, single NV center-based *T*_1_ relaxometry has emerged as a vital noninvasive experimental tool for the exploration of diverse physical properties at the nanoscale.^7,19−24^

Recently, a 2D magnetic insulator, ruthenium trichloride (α-RuCl_3_), was proposed as a potential candidate to host an exotic state of matter: a quantum spin liquid (QSL) state. In QSLs, strong quantum fluctuations inhibit long-range spin order down to very low temperatures and instead form an unconventional ground state exhibiting long-range spin entanglement and topological order.^14,25−27^ In 2006, Kitaev proposed an exactly solvable model for the QSL ground state in a honeycomb lattice, which supports the existence of fractional excitations like Majorana fermions and  gauge fluxes.^28^ Besides the exciting physics of QSLs, they also have a potential for application. For instance, long-range entangled spins have the potential to establish a quantum communication network, and Majorana fermions may serve as error-protected qubits in a future quantum computing technology.^29,30^ While α-RuCl_3_ displays intriguing magnetic phases at low temperatures, its high-temperature spin dynamics, particularly under external perturbation, is equally fascinating. For instance, the collapse of the Mott state and magnetic susceptibility has been observed at room temperature under external pressure.^31,32^ In addition, a scattering continuum, argued to be associated with fractional excitations, has been observed in room temperature polarization-resolved Raman spectra of α-RuCl_3_ single crystals.^33^ Considering the NV center’s excellent sensitivity to magnetic and electric field noise and drawing inspiration from recent theoretical proposals to employ *T*_1_ relaxometry for exploring different phases of magnetic systems,^24,34,35^ there is significant interest in utilizing NV relaxometry to explore nanoflakes of α-RuCl_3_.

In this work, we performed room temperature *T*_1_ relaxometry on single NV centers to investigate the magnetic noise originating from nanometer-thin layers of α-RuCl_3_. Our results reveal a significant reduction in *T*_1_ in the presence of these flakes. Additionally, we examined *T*_1_ under varying external magnetic fields. Our findings illustrate that external magnetic fields alter the noise profile of α-RuCl_3_ and appear to enhance the *T*_1_ of the proximate NV center.

All fluorescence experiments were performed using a custom-built confocal microscope. Illustrated in Figure 1a, a spin qubit is positioned at a distance *z* from a two-dimensional material, while Figure1b depicts an optically accessible shallow NV center qubit in a diamond nanopillar. We start out with diamond membranes with shallow single NV centers (see the Supporting Information for details); by using e-beam lithography, nanopillars that increase the photon collection efficiency are etched in the diamond. Since placing flakes on top of nanopillars is challenging, we developed a method of a hybrid bulk nanopillar diamond structure to explore atomically thin flakes using single NV centers in pillars. In this hybrid structure, we created oval-shaped regions of nanopillars covering nearly 25% of the flat diamond surface area. Hence, one can enjoy the benefits of both cases, i.e., the flat areas providing strong adhesion, helping the flakes to adhere to and encircle the nanopillars seamlessly, while the nanopillars can host a single NV center and offer high photon counts per second. An SEM image of bare diamond pillars is provided in the Supporting Information (Figure S1).

**Figure 1:**
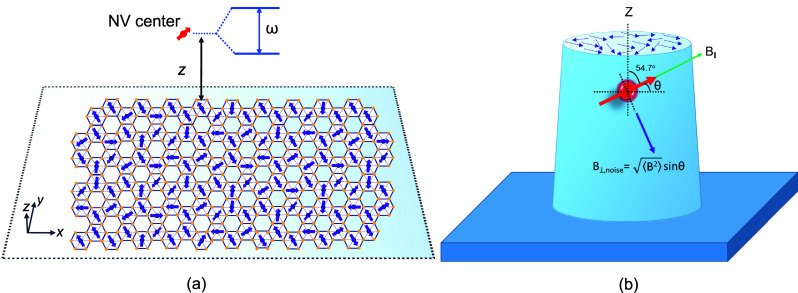
(a) Schematic of our experiment depicting a quantum sensor located at distance *z* from a 2D α-RuCl_3_ spin bath. Ruthenium trichloride acquires a monoclinic structure (space group *C*2/*m*) at room temperature, where Ru^3+^ ions form a 2D hexagonal honeycomb lattice in the *ab*-plane.^36^ (b) Depiction of the projection of 2D noise, with the external magnetic field *B*_∥_ aligned parallel to the NV quantization axis, for an NV center located in the diamond pillar.

To investigate α-RuCl_3_ using single NV center *T*_1_ relaxometry, we exfoliated thin flakes from a sufficiently large single crystal (see the Supporting Information for details on crystal growth). These flakes were then transferred onto a (100) diamond membrane patterned with nanopillars hosting NV centers using the Scotch tape dry exfoliation method. In Figure 2a, the SEM image illustrates that the transferred flake adopts a tent-like structure over the nanopillars. This pattern is similar to h-BN flakes transferred onto diamond nanopillars for artificial manipulation of curvature and strain.^37^ The thickness of the transferred flakes, as determined by atomic force microscopy (AFM), ranges from approximately 10 nm to several hundred nm. The AFM height profile of the thinnest flake (∼11 nm) examined in this study is provided in Figure S4c. Optical microscopy images reveal the thickness-dependent contrast of the transferred flakes. A relatively thick flake, approximately 100 nm in thickness, is easily distinguishable atop the pillars in the optical microscopy images, as illustrated in Figure 2c.

**Figure 2:**
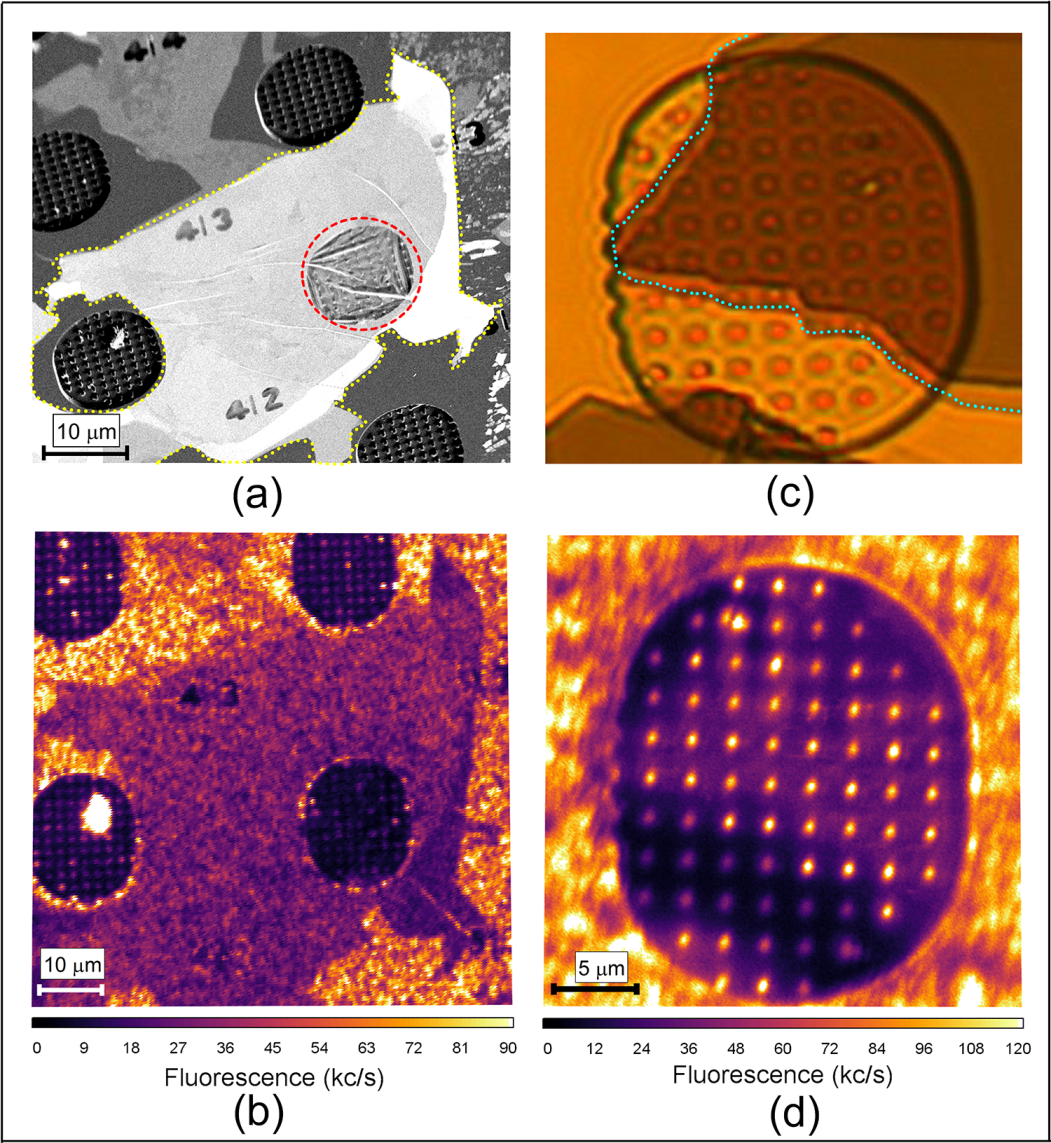
(a) A 45° tilted SEM image of one of the thin α-RuCl_3_ flakes transferred on diamond outlined by dotted yellow line; the flake forms a tent-like structure over the nanopillars (encircled by a red dashed line). (b) A confocal microscopy image of the same flake which was shown in (a). The region covered with the flake exhibits less fluorescence, indicating quenching of the fluorescence in the presence of the atomically thin flake. (c) Optical microscopy image of an ∼100 nm thick flake on the pillars, outlined by dotted line. (d) Confocal image of the ∼100 nm thick flake shown in (c). The pillars beneath the flake are brighter than the pillars outside of the flake, which is most likely due to light scattering in proximity to the flake.

In our experimental setup, we observed up to five times enhancement in the collection efficiency for nanopillars compared to flat diamond.^38−40^ A high photon collection efficiency improves sensitivity and shortens measurement times for sequences incorporating *T*_1_.^5^ Another advantage of using pillars is that they allow us to pass the laser through the bottom of the diamond to illuminate the NV centers, which minimizes the laser impact on the material on top. This becomes particularly important in the case of atomically thin materials, where the higher laser power can damage the material.^41^ Thus, the utilization of nanopillars reduces the total irradiated energy on the sample due to the combined effects of faster measurements, reduced transmission to the sample, and lower laser powers.

To explore magnetic noise from α-RuCl_3_ flakes, we performed NV center *T*_1_ relaxometry of thin flakes of α-RuCl_3_ . The NV is polarized in the spin state |*m*_*s*_ = 0⟩ using a 520 nm laser, and this state freely decays to a thermal admixture of |*m*_*s*_ = 0⟩ and |*m*_*s*_ = ±1⟩ states. The decay rate of the polarized state is dependent on the coupling of the NV center to its environment. In general, the relaxation time of the NV center in the presence of spin noise has been described as^10^
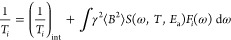
1where  refers to intrinsic relaxation mechanisms, γ is the gyromagnetic ratio of the NV center, and √⟨*B*^2^⟩ is the root-mean-square value of the effective magnetic field at the NV position produced by the proximate spin bath configuration. *E*_a_ is the energy of the magnetic anisotropy. *S*(ω) is the spectral density of the spin noise environment, and *F*(ω) is a filter function which depends on the pulse sequence used (the filter function for *T*_1_ is provided in the Supporting Information). The relaxation rate of an NV center [Γ = 1/*T*_1_], proximate to a conductive or insulating magnetic environment, is governed by the spin noise associated with the imaginary part of the magnetic susceptibility (χ″)—in other words, the imaginary part of the magnetic field autocorrelation function.^7,19,34,35,42^
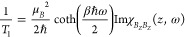
2where *z* is the distance of the noise source to the NV center and ω is the transition frequency of the NV center (the concept is schematically depicted in Figure 1a). The amplitude of the imaginary part of the magnetic susceptibility may be modified by different factors, e.g., orbital diamagnetic susceptibilities of the spinon, chargon, and diamagnetic susceptibility of the emergent gauge field.^34,35,42^ The *T*_1_ relaxation time of the NV center is susceptible to magnetic noise in the GHz regime (∼3 GHz),^10^ which is suitable for detecting the magnetic field’s autocorrelation function (magnetic noise) generated by spin liquids,^35^ metals,^43^ and magnetic insulators.^19,24^

In Figure 3b, we compare collected room temperature photoluminescence signals of a single NV center against the variation in waiting time τ in the presence and absence of ∼11 nm thick flake of α-RuCl_3_, under 1.0 ± 0.1 G and <0.5 G magnetic field condition, respectively (magnetic field is calculated from optically detected magnetic resonance). The signals show an exponential decay trend, indicating a relaxation of the NV center polarized state |*m*_*s*_ = 0⟩ to a mixed |*m*_*s*_ = 0, ±1⟩ state. The characteristic decay time *T*_1_ is calculated by fitting the signal with a stretched exponential function.^44^
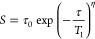
3where *S* is the signal, τ_0_ is the prefactor, τ is the waiting time, η refers to the stretching parameter, and *T*_1_ is the longitudinal relaxation time. For NV4, the fits yield *T*_1_^Ru^ = 13.2 ± 2.5 and *T*_1_ = 472 ± 20 μs with and without a flake, respectively (the fitting procedure is given in the Supporting Information), a ratio of *T*_1_/*T*_1_^Ru^ ∼ 35; such a significant reduction of *T*_1_ in the presence of a flake indicates a strong coupling of the NV spin dynamics to the magnetic noise of α-RuCl_3_. Similar reductions in *T*_1_ have been observed in the case of proximate magnetic noise sources, such as superparamagnetic Fe_3_O_4_ nanoparticles,^45^ paramagnetic Gd^3+^ spins,^46^ and ferritin nanomagnets.^10,47^ At room temperature, α-RuCl_3_ is in a paramagnetic state, confirmed by fitting the Curie–Weiss law to the inverse DC magnetic susceptibility (Figure S3). The notable reduction in *T*_1_ in the presence of α-RuCl_3_ suggests that the paramagnetic noise bath of α-RuCl_3_ couples to the NV center. The incoherent dipole–dipole interaction between the NV center and the nearby thermally fluctuating spin bath accelerates the relaxation rate of the NV center. Furthermore, in polarized Raman spectroscopy of α-RuCl_3_, a broad scattering continuum with asymmetric Fano line shapes is recorded, arising from two-dimensional magnetic scattering at room temperature.^33,48^ In general, asymmetric Fano line shapes typically originate from a coupling between discrete phonon modes and electronic states, signifying metallicity in phase-separated systems.^48−50^ Therefore, we argue that there is noise, similar to that in metals, present in α-RuCl_3_, which can account for the reduction of the *T*_1_ of a single NV center.

**Figure 3:**
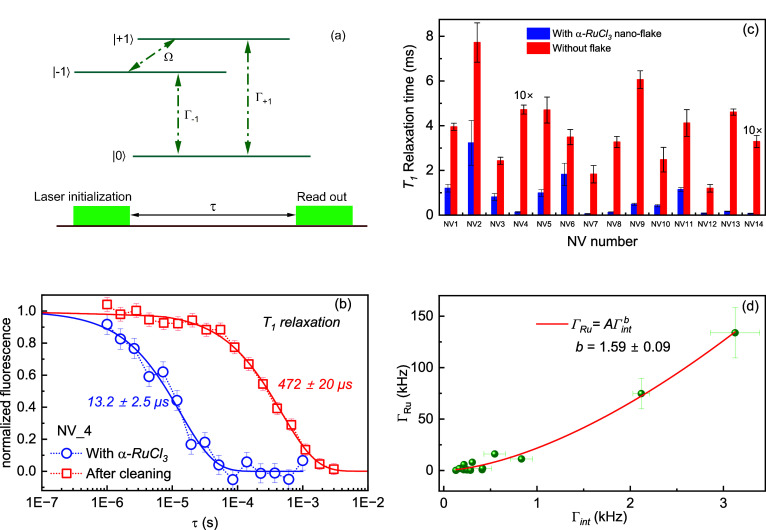
(a) Basic pulse sequence for a typical *T*_1_ measurement: The spin state |*m*_*s*_ = 0⟩ is polarized with a laser pulse. The state interacts with noise bath during the waiting time and read out by the second laser pulse. Both decay channels from |*m*_*s*_ = 0⟩ to |*m*_*s*_ = ±1⟩ are depicted in the energy diagram of the NV’s ground state shown as Γ_–1_ and Γ_+1_. Ω is related to the double quantum transition in between the |*m*_*s*_ = ±1⟩ states. (b) Comparison of the *T*_1_ relaxation time decay of a single NV center measured in the presence (blue circles) and absence (red squares) of an 11 nm thick flake of α-RuCl_3_ under 1.0 ± 0.1 G and <0.5 G magnetic fields, respectively. (c) *T*_1_ relaxation time measured for a set of single NV centers in the presence (blue) and absence (red) of thick (100 nm, NV12 to NV14) and thin flakes (∼11 nm, NV1 to NV11) of α-RuCl_3_ (external magnetic field ≤3.2 ± 0.1 G). The values of NV4 and NV14 are multiplied by a factor of 10 to make them visible on the *y*-axis. (d) Relaxation rates of NVs influenced by the α-RuCl_3_ spin bath, Γ_Ru_, plotted against their intrinsic relaxation rates Γ_int_. The solid line is a power-law fitting.

We also collected *T*_1_ data for NV centers covered by a relatively thick flake (100 nm), incorporated in Figure 3c as NV12, NV13, and NV14. The results closely resemble those observed under a thin flake. This is likely because the NV center is a highly localized probe, sensitive to its environment within 15–20 nm radius. This suggests that to investigate the thickness dependency of flakes, thickness variations should be within the dynamic range spanned by the sensing volume of the NV center. To validate this observation, we conducted *T*_1_ experiments on a total of 14 individual NV centers, both with and without the presence of flakes. The results are shown in Figure 3c, with NV1–NV11 representing thin flake conditions and NV12–NV14 under thick flake conditions. The results consistently demonstrate a reduction in *T*_1_ for each NV center in the presence of a flake.

The variation in the intrinsic *T*_1_ of the presented NVs spans approximately 1 order of magnitude, probably attributed to the variation in the depth, *d*, of the NVs. Shallower NVs exhibit shorter *T*_1_ times, and a previous study showed that in the shallow depth limit (*d* < 50 nm), the intrinsic relaxation rate was roughly inversely proportional to the NV depth, Γ_int_ ∝ *d*^–1^ (see ref  ^51^). To better understand the effect of the external Ru spin bath, we calculated the flake-induced relaxation rate according to Γ_Ru_ = [1/*T*_1 w.flake_ – 1/*T*_1 w.o.flake_]. In Figure 3d, we plotted the flake-induced relaxation rate of NVs against their intrinsic relaxation rate and found it fits well a power law Γ_Ru_ = *A*Γ_int_^*b*^ with a value of *b* = 1.59 ± 0.09. This power law dependency suggests that if one assumes the intrinsic rate is proportional to 1/*d* (see the Supporting Information for a detailed analysis), then Γ_Ru_ ∝ *d*^–1.6^. The scaling may be indicative of the physics of the 2D spin bath. Further theoretical and experimental studies, particularly with depth-calibrated NVs, may assist in revealing the source.

Investigating magnetic noise in the MHz regime, we utilized spin-echo spectroscopy (*T*_2_ measurement), and the results are illustrated in Figure S6. The experiment was conducted on the same NV center as our *T*_1_ relaxometry measurements in Figure 3b. Intriguingly, the transverse coherence time *T*_2_ is barely affected by the presence of the flake, probably due to the low noise amplitude within this specific frequency range.

To gain more insight into the magnetic behavior of the material at room temperature, we performed *T*_1_ relaxometry under the application of an external magnetic field along the quantization axis of the NV center. This method is sometimes termed cross-relaxometry.^52^ In a cross-relaxometry experiment, applying a magnetic field leads to Zeeman splitting, resulting in a linear opening of the energy gap between NV states |*m*_*s*_ = −1⟩ and |*m*_*s*_ = +1⟩. Each transition exhibits distinct decay rates, determined by the overlap of its energy with the noise spectrum of the nearby noise bath (eq 1). As a consequence, decay from the optically polarized |*m*_*s*_ = 0⟩ state exhibits the combined effect of all three decay rates, as depicted schematically in Figure 3a.

Figure 4a displays the *T*_1_ of a single NV center for two values of applied fields (2 and 383.5 G) in the presence of a flake. *T*_1_ shows a stretched exponential decay and notably increases approximately 18-fold at a higher field. In contrast, the change was negligible when we collected data for different fields without the flake (Figure 4b). In Figure 4c, we summarize our field-dependent data for *T*_1_. In the absence of the flake, the change in *T*_1_ with respect to field variation is almost negligible. Remarkably, in the presence of the flake, *T*_1_ exhibits an increase as the magnetic field strength increases, and at high field values, it nearly returns to the value observed in the absence of the flake. Such a significant increment of *T*_1_ with magnetic field at room temperature is unusual due to the incompatible energy scales, i.e., *k*_B_*T* = 25 meV and exchange interaction (*J* ∼ 3 meV),^53^ and so its origin is not immediately apparent. We discuss two possible mechanisms.

**Figure 4:**
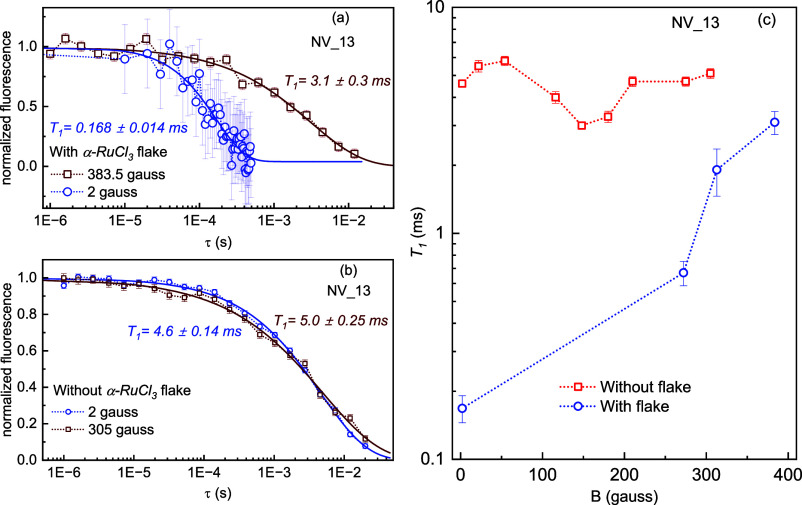
*T*_1_ magnetic field effect: Relaxation time of single NV center measured in the presence (a) and absence (b) of the α-RuCl_3_ flake for two different fields aligned parallel to the NV quantization axes. (c) *T*_1_ relaxation versus magnetic field measured in the presence (blue) and absence (red) of the α-RuCl_3_ flake.

## Diamagnetism

It has been observed that a diamagnetic electrolyte near the NV center reduces both electric and magnetic noise, consequently improving *T*_1_.^54^ A theoretical calculation for the *T*_1_ relaxation time of an NV center proximate to a 2D spin liquid shows similar effects of a diamagnetic state on the *T*_1_.^34^ It is worth noting that a Curie–Weiss fit of the magnetic susceptibility gives a magnetic moment of 2.31 μ_B_. The inverse susceptibility data are fitted with a Curie–Weiss formula at a high-temperature regime to calculate the total paramagnetic moment (Figure S3). This value is significantly larger than the spin-only contribution of Ru^3+^ in its low-spin state, which is 1.73 μ_B_,^55^ and indicates a significant contribution from the orbital moment. Interestingly, the calculated orbital moment is nearly one-third of the spin moment, resembling the free electron model, where the Landau diamagnetic response of the orbitals is precisely one-third of the Pauli paramagnetic response.^56^ In their recent theoretical work, Banerjee and Lin ^57^ explored the intertwining of spin and orbital magnetization in α-RuCl_3_ using the multiorbital spin–orbit model for Mott insulators such as α-RuCl_3_. They formulate that local current loops are induced when an external magnetic field is applied to such Mott insulators. This generates magnetization opposite to the applied field as a diamagnetic response similar to Landau diamagnetism in metals. Because of the substantial orbital moment present in α-RuCl_3_, we propose that applying an external magnetic field could induce a diamagnetic-like response in α-RuCl_3_, potentially leading to an enhancement in *T*_1_.

## Charge Redistribution

In a recent work, a noticeable enhancement in the transverse coherence time of a NV center has been observed in the presence of a superconductor.^58^ The effect is tentatively rationalized as a change in the electric noise due to the superconductor (diamagnetic)-induced redistribution of charge carriers near the NV center. α-RuCl_3_ is a small band gap semiconductor with room temperature resistivity of the order of 10^–1^ Ω cm.^59^ In α-RuCl_3_, due to the onsite Coulomb repulsion, a gap opens within the Ru 4d band and splits it into two sub-bands, the upper Hubbard band and the lower Hubbard band, and an intersite d^5^d^5^ → d^4^d^6^ charge transfer between neighboring Ru^3+^ ions governs the hopping conduction mechanism.^60^ Along parallel lines as those of the change in transverse coherence time due to charge fluctuations, we therefore suggest that these could be an additional source of noise at room temperature. Charge fluctuations are sensitive to external perturbations such as magnetic fields, especially in poor conductors. An external magnetic field may reduce the mobility of charges, leading to a decrease in charge fluctuations, and could enhance the *T*_1_. The electrical conductivity of single-crystal α-RuCl_3_ is highly anisotropic, with a reported conductivity almost 4 orders of magnitude higher in a plane perpendicular to the *c*-axis compared to the plane parallel to the *c*-axis.^61^ The longitudinal relaxation time of an NV center is sensitive to the noise projected perpendicular to its quantization axis. In the presence of magnetic noise coupled to an NV center, the longitudinal relaxation rate can be expressed as^62^
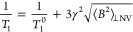
4where γ = 28 GHz/T is the electron gyromagnetic ratio and *B* is the magnetic field noise component perpendicular to the NV quantization axis. The schematic of the experimental geometry is shown in Figure 1b. The noise bath comprising electronic spins and charge fluctuation lies in the *ab*-plane. The component of this noise perpendicular to the quantization axis of the NV center will be
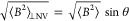
5where θ is the angle between the noise bath and NV quantization axis. Considering the two-dimensional nature of the spin–spin interactions in the hexagonal *ab*-plane, we believe that applying the magnetic field parallel to the NV quantization axis will diminish the angle θ and the noise component perpendicular to the NV quantization. This suggestion further validates our observation of *T*_1_ recovery at a high field.

In conclusion, we have investigated nanometer thick flakes of α-RuCl_3_ using the longitudinal spin relaxation time *T*_1_ of single NV centers in diamond. We observed a significant reduction of the NV relaxation time by the proximity of α-RuCl_3_, which signifies the coupling of noise fluctuations of α-RuCl_3_ with the NV. A gradual increment of *T*_1_ time as a function of magnetic field in the flake’s presence was observed. We hypothesize that the increase in *T*_1_ is associated with the suppression of spin and charge fluctuations in atomically thin flakes due to the magnetic field. These results indicate that *T*_1_ relaxometry with a single NV center is a promising technique to explore the spin dynamics of nanoflakes of the potential quantum spin liquid candidate α-RuCl_3_ and similar systems. A comprehensive low-temperature *T*_1_ relaxometry study, along with supporting experiments such as magnetoresistance, is required to corroborate our interpretation. We also propose exploiting a (111) oriented scanning NV center to study the out-of-the-plane spin dynamics confined in the hexagonal *ab*-plane.^63^ Furthermore, unlike neutron scattering in iridium-containing compounds, the NV qubit does not suffer from issues related to flux absorption and the amount of the sample, rendering it a desirable sensor for the family of iridium-based QSLs.

## Data Availability

The data that support the findings of this study are openly available at the following URL/DOI: https://doi.org/10.34933/a374143b-7a63-4264-88f0-51beecdf1356.

## Supporting Information Available

Diamond membrane, pillars, and implantation of NV centers; verification of single emitters in the NV centers; single crystal synthesis; atomic force microscopy; filter function for longitudinal relaxation time; *T*_2_ dephasing spectroscopy; DC magnetic susceptibility; data normalization process; fitting of *T*_1_ relaxation times; fitting parameters; NV relaxation dependence on depths (PDF)
