# Supplementary material for: Room Temperature Relaxometry of Single Nitrogen Vacancy Centers in Proximity to α-RuCl3 Nanoflakes
Source: Nano Lett. 2024 Apr 8;24(16):4793–800. doi: 10.1021/acs.nanolett.3c05090 (PMC11057446; doi:10.1021/acs.nanolett.3c05090)
Supplement: Supplementary file 1 — nl3c05090_si_001.pdf [file nl3c05090_si_001.pdf]

Room temperature relaxometry of single nitrogen-vacancy centers in proximity to α-RuCl$_3$ nanoflakes Supplementary Information

Jitender Kumar,[†] Dan Yudilevich,[†] Ariel Smooha,[†] Inbar Zohar,[†] Arnab K. Pariari,[‡] Rainer Stöhr,[¶] Andrej Denisenko,[¶] Markus Hücker,[‡] and Amit Finkler[*,†]

†*Department of Chemical and Biological Physics, Weizmann Institute of Science, Rehovot, Israel*

‡*Department of Condensed Matter Physics, Weizmann Institute of Science, Rehovot, Israel*

¶*3rd Institute of Physics, IQST and ZAQuant, University of Stuttgart, 70569 Stuttgart, Germany*

E-mail: amit.finkler@weizmann.ac.il

Diamond membrane, pillars and implantation of NV centers

In this study, we utilized a single crystal [100] diamond membrane with dimensions [$2\times2\times0.03$] mm, grown using chemical vapor deposition (CVD). NV centers were created by implanting the membrane with $^{15}N^+$ nitrogen ions with an energy of 9.8 keV and afterwards annealing in high vacuum at a temperature of 950°C for two hours. Due to straggle/channeling effects

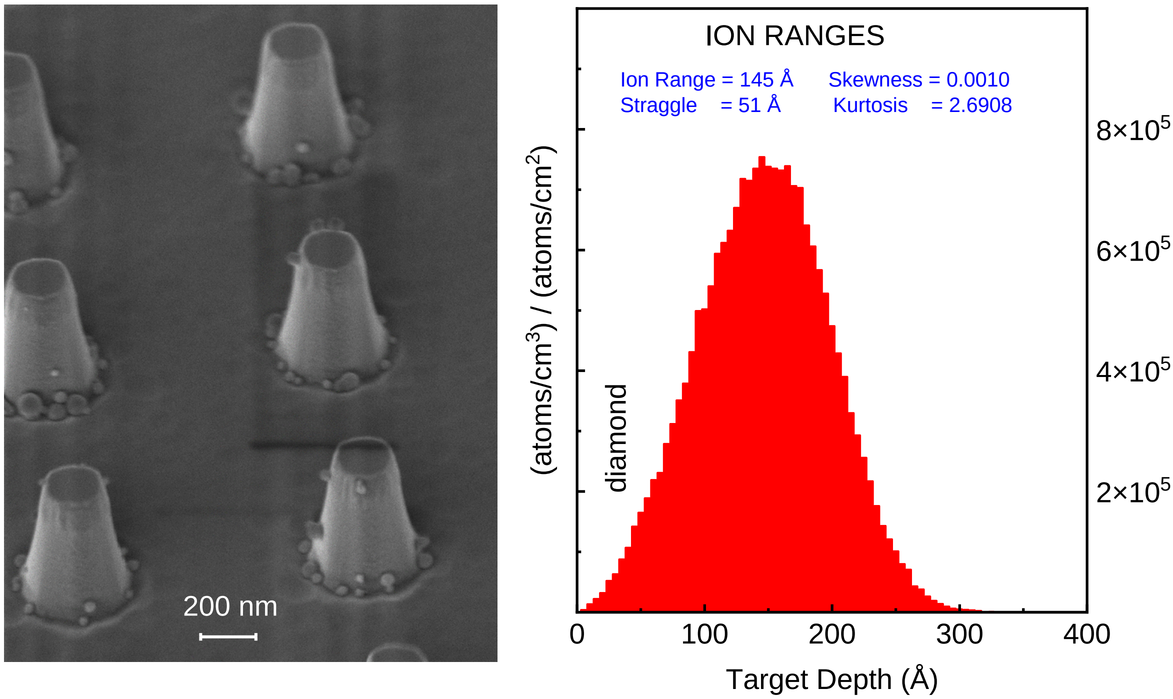

Figure S1: On the left, 45 degree tilted SEM image of diamond nano-pillars. On the right, SRIM simulation for depth profile of implanted NVs at 9.8 keV energy.

during ion implantation, the NV depths of implanted shallow NVs can vary quite a lot, a Stopping and Range of Ions in Matter (SRIM)[1] simulation is shown on the right of Fig. S1. The corresponding depth of the implanted NVs is around 14±5 nm. An SEM image of nanopillars etched in the implanted diamond are shown in the left of Fig. S1.

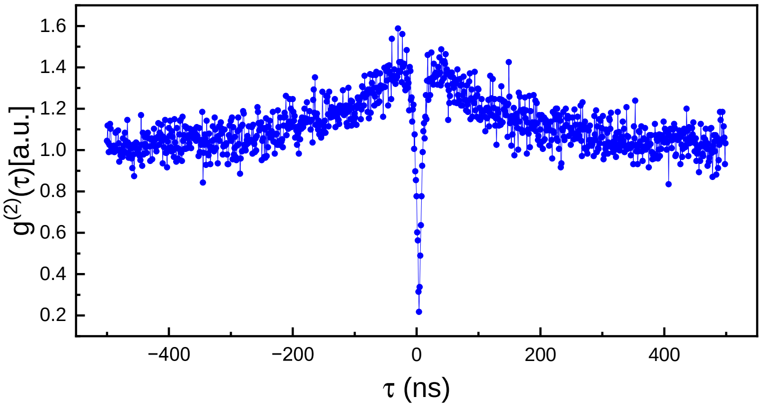

Figure S2: $g^2(\tau)$ measurement of NV_3 on a diamond nanopillar situated under the thin (11 nm) α-$RuCl_3$ flake.

Verification of single emitters in the NV centers

To find out whether the pillars below the flakes host a single NV defect or not, we recorded the second-order photon autocorrelation function $g^{(2)}(\tau)$. We find $g^{(2)}(\tau) < 0.5$ for $\tau \to 0$, which is an indication for the detection of a single photon emitter[2]. Figure S2 illustrates the results of $g^2(\tau)$ measurements on NV_3 in a nanopillar positioned beneath the thin flake. In this study, we performed measurements on fourteen different single NVs. We selected the NVs with reasonable contrast (above 10%) and T > 100 µs.

Single crystal synthesis

Single crystals of α-$RuCl_3$ were obtained in three steps starting from commercially available ruthenium (III) chloride powder, which is anhydrous and contains a minimum of 47.7% ruthenium (Alfa Aesar). The process involves the dehydration, transformation of the α-$RuCl_3$ and β-$RuCl_3$ phase mixture in commercial ruthenium (III) chloride powder into the α-$RuCl_3$ phase, and, finally, single crystal growth from polycrystalline α-$RuCl_3$ using the vapor transport technique (VT)[3–6]. Dehydration (for any residual water vapor) involved baking of ruthenium (III) chloride powder at 250^0 C for 48 hours under continuous Ar flow (4N purity) in an air-sealed quartz tube arrangement, followed by rapid cooling to room temperature. After this process, the sample was transferred to an argon glove box for further processing.

About 0.5 g of the powder sample was sealed in a 27 cm long and 16 cm inner diameter quartz ampule under vacuum (10^{-5} mbar). The sealed ampule was placed inside a larger diameter quartz ampule within a two-zone temperature gradient furnace, and continuous Ar flow provided additional protection. Initially, both temperature zones of the furnace were set to 600°C and held there for 24 hours. In this stage phase-pure α-$RuCl_3$ was obtained as β-$RuCl_3$ in the initial powder mixture transforms irreversibly into the α-$RuCl_3$ phase above 395^0C.

Then, the temperature at the sample end of the sealed quartz ampule was set to 800° C, and the temperature at the other end was set to 720°C, and this configuration was maintained for 48 hours. Our intention was to gather all the materials at one end of the ampule before initiating the actual vapor transport (VT) process that stimulates crystal growth. After 48 hours, the temperature gradient was reversed, positioning our poly-crystalline powder sample at the hot end. After 7 days, the hot zone was gradually cooled to 650°C, and the cold zone was similarly cooled to 600°C at a very slow rate of 2°C per hour. Finally, the ampule was air-quenched by rapidly taking it outside the furnace. Thin flake-like crystals with dimensions in the range of a few millimeters were obtained from the cold end of the tube. The initial dc magnetic characterization was conducted using a 7 T Superconducting Quantum Interference Device (SQUID) Magnetic Properties Measurement System (Quantum Design).

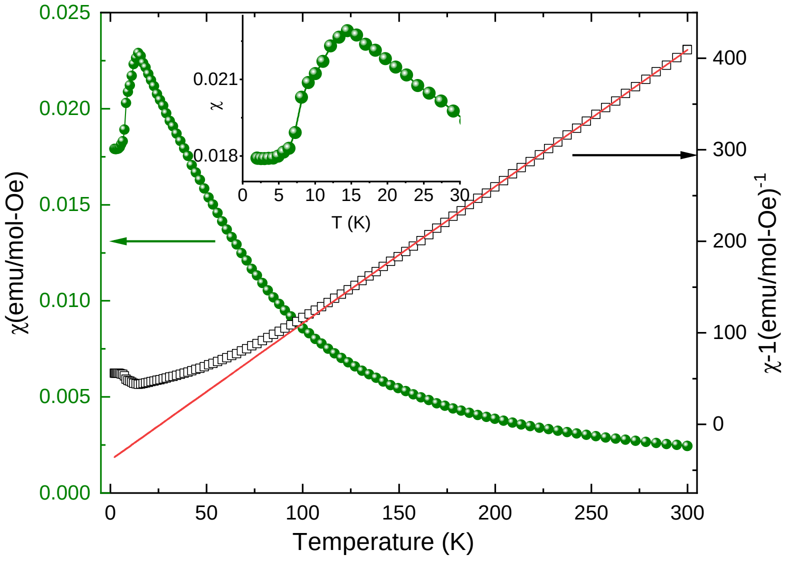

Figure S3: In the left panel, depicts the temperature dependence of dc magnetization as measured in α-$RuCl_3$ in the zero-field-cooled (ZFC) protocol under 500 Oe magnetic field. The inset shows a Curie-Weiss fit in the high-temperature paramagnetic region. The right panel shows a Curie-Weiss fit (red line) on the inverse susceptibility in the high-temperature paramagnetic region. The inset shows the zoomed section of the measurement near the phase transition temperatures. Two transitions are visible at 14 K and 7 K.

Atomic force microscopy

The exfoliated flakes transferred onto the diamond were initially examined using an optical microscope. Subsequently, their thickness was estimated by atomic force microscopy. The AFM image and the height profile of one of the thinnest flakes, which formed a tent-like shape over the nanopillar area circled in Fig. 2 (main text), are depicted in Figs. S4 (a) and (b), respectively.

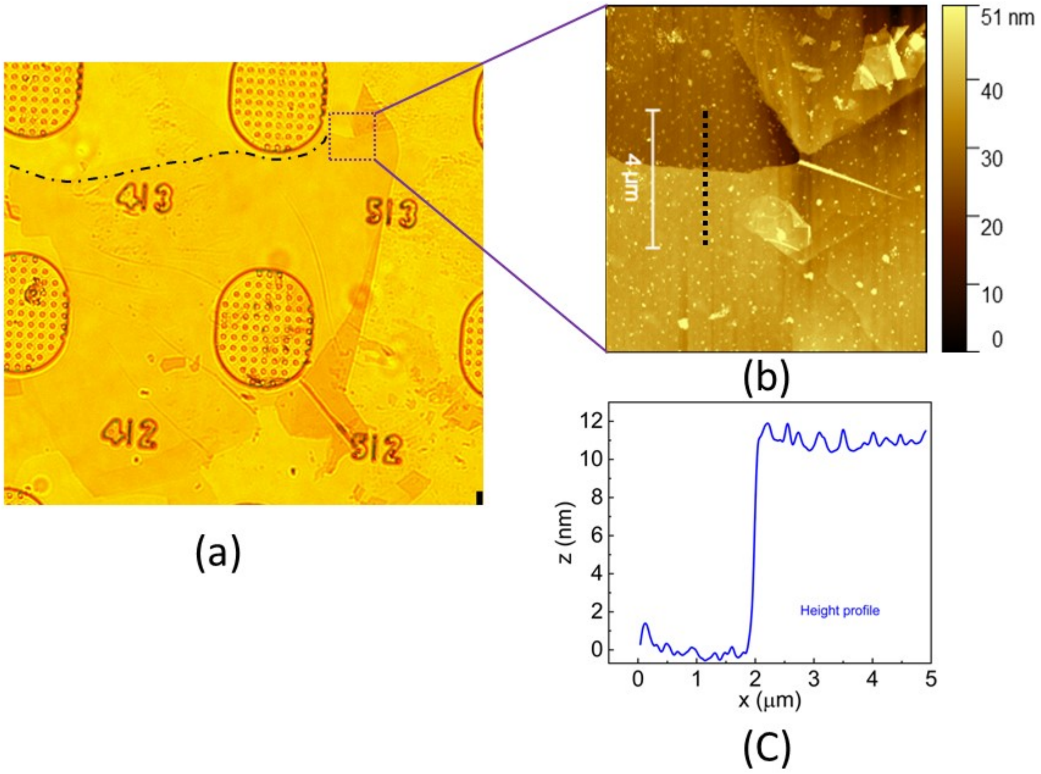

Figure S4: (a) An optical microscope image of a thin flake transferred onto the diamond, used in this study. Dashed dot line depicting one edge of the flake. The area depicted in the dashed square was used to record an AFM image and height profile. (b) The AFM image displays one corner of the transferred flake on the nanopillars, the same flake featured in the SEM image shown in the main text. The dashed black line indicates the cross-section from which the height profile of the flake was taken (c) The height profile of the same flake, shown on the left side, indicates a thickness of approximately 11 nm.

.

The filter function for longitudinal relaxation time

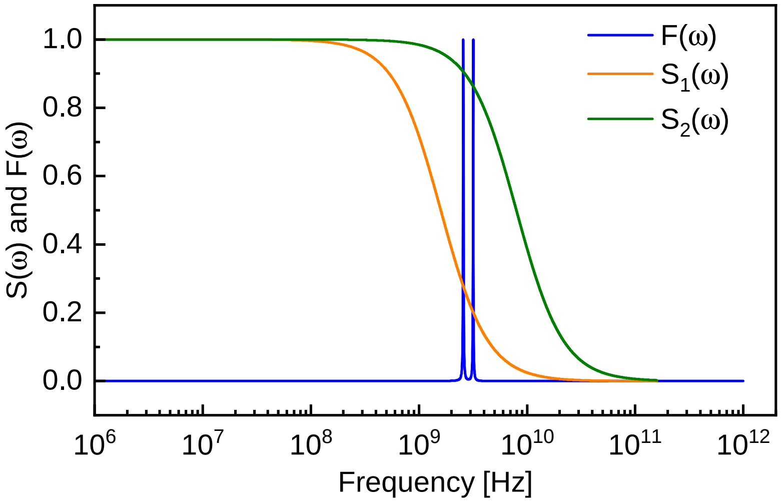

Figure S5: Spectral density and filter function associated with a T_1 experiment of NV center in frequency domain (normalized to one). $S_1(\omega)$ represents magnetic fluctuations with a relaxation frequency of 10 GHz and $S_2(\omega)$ - 50 GHz.

The filter function for T_1 relaxometry is given by

$$F(\omega) = \frac{1}{\pi}\frac{1/T_2^*}{(1/T_2^*)^2 + (\omega - \omega_{+1})^2} + \frac{1}{\pi}\frac{1/T_2^*}{(1/T_2^*)^2 + (\omega - \omega_{-1})}$$

where $T_2{}^*$ is the Ramsey dephasing time and $\omega_{\pm 1}$ are the microwave transition frequencies for m_s=0 $\rightarrow$ m_s=$\pm$1 spin states. In Fig. S5, we plotted the typical filter function $F(\omega)$ for the longitudinal relaxation process of the NV center, including two single quantum transitions in the GHz regime (depends on the applied magnetic field). $S_1(\omega)$ and $S_2(\omega)$ are the two different power spectral densities of a noise bath; the spectral width and amplitude of $S_1(\omega)$ and $S_2(\omega)$ depend on several factors like temperature, the thickness of the flake (bath nature), external applied magnetic and electric fields, and pressure. The decay rate of the NV center is governed by the overlap of the filter function with the noise bath's power spectral density, as mentioned in Eq. 1 in the main text.

T_2 dephasing spectroscopy

In Fig. S6, the results of Hahn-echo spectroscopy with and without flake are shown, performed on the same NV center for which T_1 relaxometry is depicted in Fig. 3b in the main text. The presence of the flake has a negligible effect on the transverse coherence time T_2.

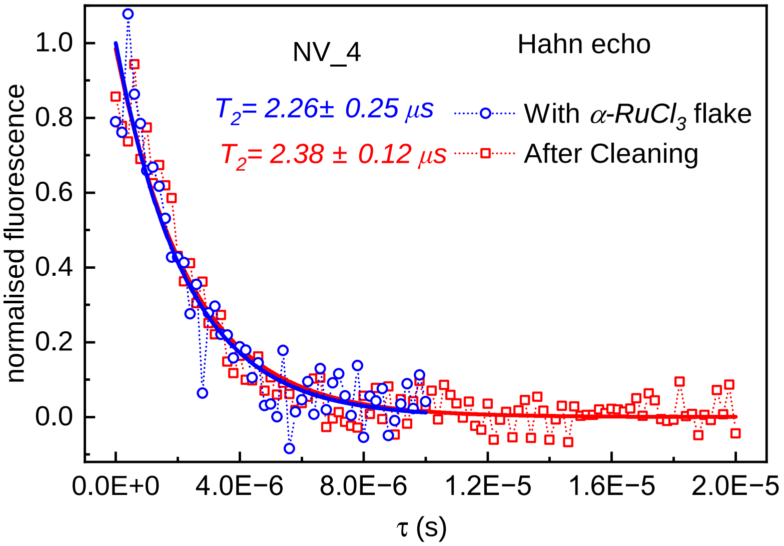

Figure S6: Hahn-echo experiment with and without 11 nm flake. Solid lines are the exponential fit on the data; fit yields the value 2.26 ± 0.25 μs and 2.38 ± 0.12 μs with and without flake respectively.

DC magnetic susceptibility

We performed temperature-dependent magnetic measurements of one large single crystal to explore the magnetic behavior of the grown single crystals (Fig. S3. With a step, a clear Λ-shaped feature is observed in zero-field-cooled data. We zoomed in on the feature in the inset, and two successive magnetic transitions appeared at 14 K and 7 K, corroborating with the published results[7]. To calculate the paramagnetic spin moment of Ru^{3+} A Curie-Weiss fit to the inverse dc magnetic susceptibility in the high-temperature paramagnetic region, as shown in the inset of Fig. S3.

Data normalization process

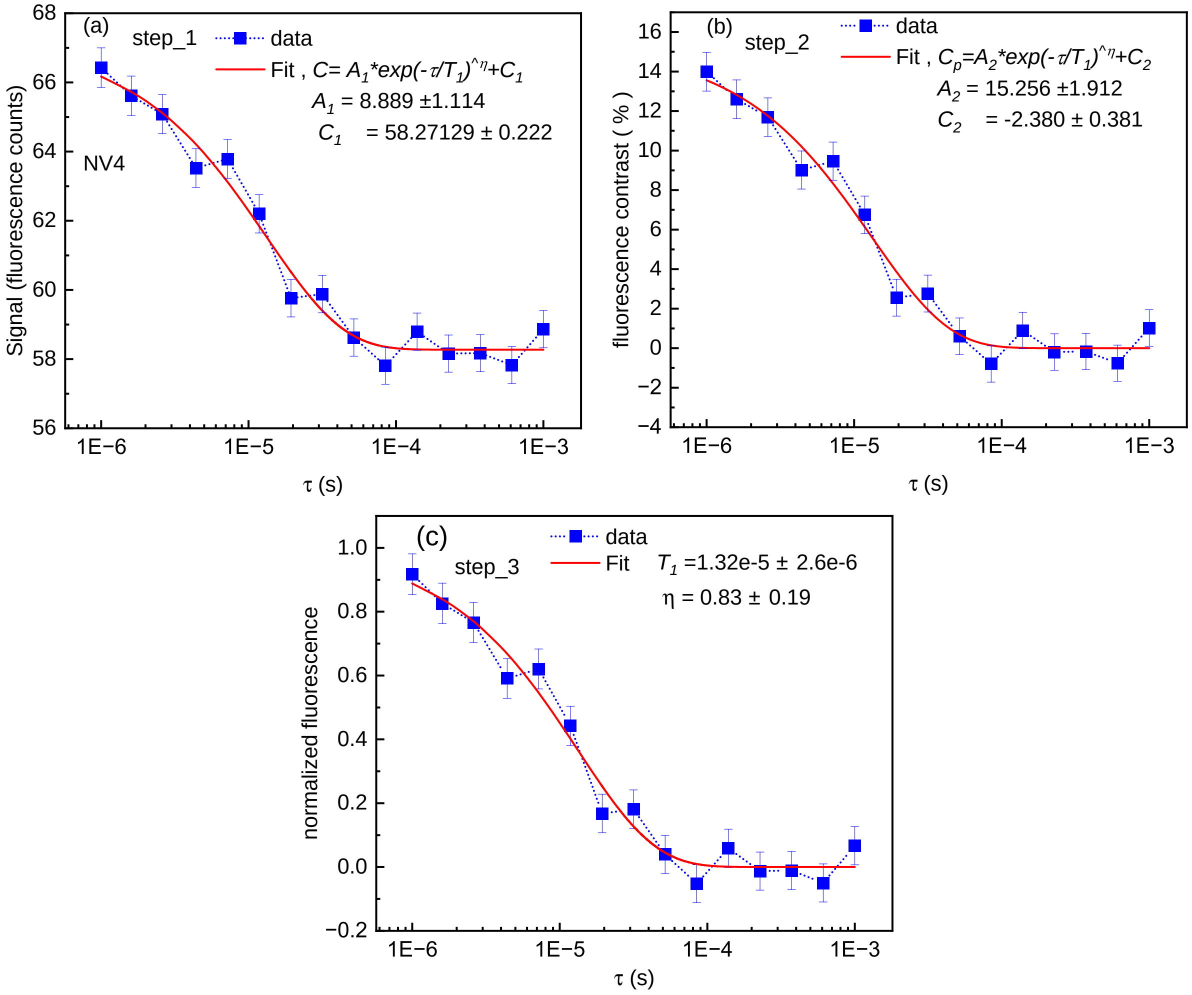

Figure S7: (a) Photon counts on the y-scale are plotted against variations in waiting time. The solid line depicts a non-exponential fit to obtained parameters A_1 and C.(b) Percentage contrast variation against the waiting time τ. The solid line depicts a non-exponential fit to obtained parameters A_2 (c) Normalized data is plotted and fitted with a non-exponential fit expression.

In T_1 and T_2 measurements, we collected the photoluminescence data in the presence and absence of flakes and observed variations in contrast in both runs. For better representation, it is essential to normalize the fluorescence data and present them on the same scale. In this work, we re-scaled the photoluminescence contrast value on a scale from 1 to 0. In our

experiments, we recorded the variation of waiting time τ and collected photon counts from the APDs. The scaling process involved three steps, which are explained below:

1. We fitted the collected raw data (photon counts, c) with a non-exponential decay expression, as illustrated in Fig. S7(a).

$$c = A_1 \exp\left(-\frac{\tau}{T_1}\right)^{\eta} + C_1,$$

   where, τ, T_1 and η represent the waiting time, longitudinal relaxation time, and stretched exponent, respectively. The parameter A_1 contains the information about the photon count difference between two states (fully polarized to a mixed state). The parameter C is related to photon counts in a fully mixed state at equilibrium ($\tau \to \infty$).

2. In this step, we converted the photoluminescence into a percentage contrast variation against the waiting time τ, shown in the Fig. S7(b). This was achieved by using the expression below:

$$c_p = 100\left[\left(c - C_1\right)/C_1\right],$$

   After this conversion, we proceeded to fit the data once more using the following expression.

$$c_p = A_2 \exp\left(-\frac{\tau}{T_1}\right)^{\eta} + C_2,$$

   From this fit, we note the fitting parameter A_2; it will be needed in the next step.

3. In this step, we set the $\tau = 0$ limit to 1 by dividing the value obtained in the previous step by parameter A_2.

We applied a stretched exponential fit on the re-scaled data for process validation, data with the fitting depicted in Fig. S7(c), by fixing $\tau \to \infty$ to zero.The data profile and cru-

cial fitting parameters like T_1 and η remained unchanged confirming the reliability of the normalization procedure.

Fitting of T_1 relaxation time

Since this is the supplementary information, we allow ourselves to digress from the main text and delve a bit deeper into the origins of relaxation in a more pedagogic way - all of that in order to better understand the stretched exponential decay of T_1 we observe in the system. In a physical system, relaxation time is the system's transformation rate from its non-equilibrium or transient state to an equilibrium state when subjected to external perturbations such as pressure, magnetic, electric fields, and temperatures. In case of the NV center, T_1 relaxation time is the decay rate of its polarized spin state $m = 0$ to a thermal admixture of 0 and ± 1 states. Relaxation is generally described by the function[8]:

$$P(t) = P_\infty + P_0 \cdot \phi(t)$$

P(t) is a physical parameter, P_∞ and P_0 are the constants, and $\phi(t)$ is the normalized relaxation function. The well-known relaxation function is a simple exponential function:

$$\phi(t) = e^{(-t/\tau)}$$

where τ is the characteristic relaxation time of the system. However, a simple exponential relaxation is rarely found in nature. It is typically observed in systems in which interactions are weak. The Fourier transform of a simple exponential decay yields a Lorentzian peak profile without additional broadening or asymmetry, indicating a single relaxation process at play. For example, exponential dielectric relaxation is generally limited to water in a liquid state and very dilute solutions of polar liquids in non-polar solvents. In physical systems characterized by a wide distribution of relaxation times due to spatial and temporal

inhomogeneity, simple exponential laws do not adequately describe the relaxation behavior. In 1854, R. Kohlrausch introduced a non-exponential decay model known as the "stretched exponential" to characterize the charge of a capacitor, represented as[9]:

$$\phi(t) = e^{(-t/\tau)^{\eta}}$$

where η is an empirical parameter with a value between 0 and 1 for a stretched exponential and greater than 1 for a compressed exponential. Subsequently, in 1970, G. Williams and D. C. Watts utilized the stretched exponential to describe the broadening and asymmetry in dielectric spectra of polymers, transforming it into the Kohlrausch–Williams–Watts (KWW) function[10]. The latter is close to the Havriliak–Negami function in frequency domain, which accounts for the broadening and asymmetry of the relaxation peak observed in heterogeneous systems (spatial and temporal heterogeneity)[11,12]. Numerous attempts have been undertaken to elucidate the stretched exponential behavior by representing it as a linear superposition of simple exponential decay functions[13,14].

Empirical stretched exponential decay or KWW function are widely used to explain the dynamics in various areas of science, from polymers, glasses, glass-forming liquids, spin-glasses, luminescence decay of colloidal quantum dots, NV centers in nano-diamonds to relaxation processes in dipole-coupled nitrogen-vacancy centers[15–20].

The relaxation rate of an NV center proximate to the randomly fluctuating magnetic with a single correlation time τ_c is stated as[21]:

$$\Gamma_1^{\text{ext}} = 3\gamma_e^2 B_\perp^2 \frac{\tau_c}{(1 + \omega_0^2 \tau_c^2)},$$

where γ_e is the electron gyromagnetic ratio and $B_\perp^2$ is the variance of the fluctuating magnetic field in the direction transverse to the NV quantization axis. Due to a single correlation time, this expression supports a single exponential decay.

In the present case, assuming the noise bath consists of several components like spin and

charge fluctuations in α-RuCl$_3$ and P1 centers, there is a possibility that multiple characteristic relaxation times could exist for such a noise bath. Our observations indicate that our T_1 measurements align effectively with the non-exponential decay rate. We believe that the non-exponential decay is due to the compound effect of different relaxation time scales of thermal fluctuations of the proximity noise bath of α-RuCl$_3$ and intrinsic spins of the P1 centers. The table-1 presents the parameters obtained from non-exponential fitting of T_1 measurements.

Table-1

NV label	T_1	η
NV4 (with flake)	13.2 ±2.5μs	0.83 ±0.19
NV4 (without flake)	472 ±20 μs	0.81 ±0.04
NV13 (2 Gauss with flake)	0.168 ±0.014 ms	1.22±0.23
NV13 (383.5 Gauss with flake)	3.1 ±.30 ms	0.62±0.05
NV13 (8 Gauss without flake)	4.6±0.15 ms	0.64±0.02
NV13 (305 Gauss without flake)	5.0±0.25 ms	0.53±0.02

The main text discussed the results and patterns of the single NV center T_1 times observed both in the presence and absence of α-RuCl$_3$ flakes and the influence of an external magnetic field. The occurrence of a stretched exponential decay η is exciting and hence discussed briefly here. For instance, in the case of NV4, η remains approximately constant in both conditions—with and without the flake—suggesting that the observed η is primarily associated with intrinsic mechanisms, such as P1 centers. In the case of NV13, η did not change significantly under an external magnetic field when there was no flake on top of it.

Interestingly, the maximum variation in η is observed when the magnetic field changes in the presence of a flake, suggesting an external magnetic field alters the relaxation dynamics or correlation time. A careful study of the different mechanisms for the resulting stretched exponential decay we observe lies outside the scope of this work and requires further study.

Fitting parameters

The fitting parameters obtained from the fitting of NVs decay time shown in Fig. 3c of the main text are tabulated in Table 2 below.

Table-2

NV label	with flake	without flake
NV1	stret. exp. $T_1 = 1.20 \pm 0.17\,\text{ms}$<br>$\eta = 1.40 \pm 0.39$	stret. exp. $T_1 = 3.95 \pm 0.16\,\text{ms}$<br>$\eta = 0.92 \pm 0.04$
NV2	exp. $T_1 = 3.23 \pm 1.00\,\text{ms}$	exp. $T_1 = 7.72 \pm 0.88\,\text{ms}$
NV3	stret. exp. $T_1 = 0.81 \pm 0.15\,\text{ms}$<br>$\eta = 0.69 \pm 0.13$	stret. exp. $T_1 = 2.43 \pm 0.16\,\text{ms}$<br>$\eta = 0.86 \pm 0.07$
NV4	stret. exp. $T_1 = 13.20 \pm 2.5\mu\text{s}$<br>$\eta = 0.83 \pm 0.19$	stret. exp. $T_1 = 472 \pm 20\mu\text{s}$<br>$\eta = 0.81 \pm 0.04$
NV5	exp. $T_1 = 0.99 \pm 0.15\,\text{ms}$	stret. exp. $T_1 = 4.7 \pm 0.58\,\text{ms}$<br>$\eta = 0.98 \pm 0.16$
NV6	exp. $T_1 = 1.82 \pm 0.50\,\text{ms}$	stret. exp. $T_1 = 3.49 \pm 0.34\,\text{ms}$<br>$\eta$=0.94±0.12
NV7	stret. exp. $T_1 = 0.060 \pm 0.007\,\text{ms}$<br>$\eta = 0.84 \pm 0.15$	exp. $T_1 = 1.83 \pm 0.39\,\text{ms}$
NV8	exp. $T_1 = 0.120 \pm 0.024\,\text{ms}$	stret. exp. $T_1 = 3.27 \pm 0.025\,\text{ms}$<br>$\eta = 0.65 \pm 0.04$
NV9	stret. exp. $T_1 = 0.48 \pm 0.05\,\text{ms}$<br>$\eta = 1.9 \pm 0.5$	exp. $T_1 = 6.06 \pm 0.40\,\text{ms}$
NV10	exp. $T_1 = 0.42 \pm 0.05\,\text{ms}$	exp. $T_1 = 2.48 \pm 0.55\,\text{ms}$
NV11	stret. exp. $T_1 = 1.14 \pm 0.09\,\text{ms}$<br>$\eta = 1.1 \pm 0.1$	stret. exp. $T_1 = 4.12 \pm 0.60\,\text{ms}$<br>$\eta = 1.20 \pm 0.28$
NV12	exp. $T_1 = 0.083 \pm 0.009\,\text{ms}$	stret. exp. $T_1 = 1.20 \pm 0.17\,\text{ms}$<br>$\eta = 0.41 \pm 0.03$
NV13	stret. exp. $T_1 = 0.168 \pm 0.01\,\text{ms}$<br>$\eta = 1.12 \pm 0.14$	stret. exp. $T_1 = 4.61 \pm 0.14\,\text{ms}$<br>$\eta = 0.64 \pm 0.01$
NV14	exp. $T_1 = 7.30 \pm 1.30\mu\text{s}$	exp. $T_1 = 0.32 \pm 0.02\,\text{ms}$

The NV relaxation dependence on depth

Relaxation processes (namely, T_1, T_2) of shallow single NV centers have been shown to have a direct dependence on the NV depth, i.e., the distance from the diamond's surface[22]. This is due to the major impact of surface spin and charge noise. Shallow in this context means up to a few tens of nanometers deep.

To our knowledge, the dependence of T_1 on NV depth does not appear in the literature. However, in a study focused on decoherence (T_2), Myers et al.[22] also measured the T_1 of depth-calibrated NV centers. Here, we analyzed their data for shallow NV centers (up to 50 nm, in Fig. 2c) to determine the scaling of T_1 on the depth of shallow NV centers. The analysis is shown in Fig. S8. we fit the data to a power law model - $T_1(d) = Ad^b$, and find that is fits an exponent of $b = 1.06 \pm 0.22$. From this, we deduce that for shallow NVs the T_1 is generally proportional to the NVs depth. We note that the range of NV depths and T_1 values in this data also fit those measured in this work, and this data was collected on NVs in a bulk diamond (as opposed to nanodiamond crystals). The specific material

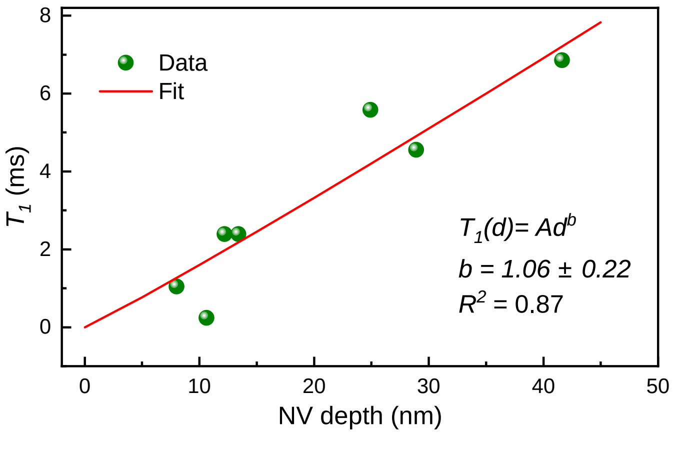

Figure S8: The dependence of shallow NV T_1 on depth, fit to a power law. The data was reproduced from Fig. 2c of Myers et al.[22].

processing of the diamond and surface treatments[23] may also impact, and we expect these details to be embodied in the coefficient A. At the same time, the scaling would depend on

the physical mechanisms responsible for the longitudinal relaxation of the NV spin. A more general picture should acknowledge the saturation of T_1 at greater depths. A simple model, for example, would be of the form - $T_1\left(d\right) = \left(\Gamma_{bulk} + \Gamma_{surf}\left(d_0/d\right)^b\right)^{-1}$. However, in the limit of shallow NVs ($d \ll d_0$), as can be seen in Fig. S8, the power law model fits well.

References

(1) Ziegler, J. F.; Biersack, J. P. The Stopping and Range of Ions in Matter. **1985**, 93–129.

(2) Jelezko, F.; Wrachtrup, J. Single defect centres in diamond: A review. *Phys. Status Solidi A* **2006**, *203*, 3207–3225.

(3) Hasegawa, Y.; Aoyama, T.; Sasaki, K.; Ikemoto, Y.; Moriwaki, T.; Shirakura, T.; Saito, R.; Imai, Y.; Ohgushi, K. Two-phonon absorption spectra in the layered honeycomb compound α-$RuCl_3$. *J. Phys. Soc. Jap.* **2017**, *86*, 123709.

(4) Leahy, I. A.; Pocs, C. A.; Siegfried, P. E.; Graf, D.; Do, S.-H.; Choi, K.-Y.; Normand, B.; Lee, M. Anomalous thermal conductivity and magnetic torque response in the honeycomb magnet α-$RuCl_3$. *Phys. Rev. Lett.* **2017**, *118*, 187203.

(5) Baek, S.-H.; Do, S.-H.; Choi, K.-Y.; Kwon, Y. S.; Wolter, A. U. B.; Nishimoto, S.; van den Brink, J.; Büchner, B. Evidence for a field-induced quantum spin liquid in α-$RuCl_3$. *Phys. Rev. Lett.* **2017**, *119*, 037201.

(6) Lang, F.; Baker, P. J.; Haghighirad, A. A.; Li, Y.; Prabhakaran, D.; Valentí, R.; Blundell, S. J. Unconventional magnetism on a honeycomb lattice in α-$RuCl_3$ studied by muon spin rotation. *Phys. Rev. B* **2016**, *94*, 020407.

(7) Banerjee, A. et al. Proximate Kitaev quantum spin liquid behaviour in a honeycomb magnet. *Nat. Mater.* **2016**, *15*, 733–740.

(8) Lukichev, A. Physical meaning of the stretched exponential Kohlrausch function. *Phys. Lett. A* **2019**, *383*, 2983–2987.

(9) Kohlrausch, R. Theorie des elektrischen Rückstandes in der Leidener Flasche. *Ann. Phys.* **1854**, *167*, 179–214.

(10) Williams, G.; Watts, D. C. Non-symmetrical dielectric relaxation behaviour arising from a simple empirical decay function. *Trans. Faraday Soc.* **1970**, *66*, 80–85.

(11) Havriliak, S.; Negami, S. A complex plane representation of dielectric and mechanical relaxation processes in some polymers. *Polymer* **1967**, *8*, 161–210.

(12) Havriliak, S.; Negami, S. A complex plane analysis of α-dispersions in some polymer systems. *J. Polym. Sci.* **1966**, *14*, 99–117.

(13) Johnston, D. C. Stretched exponential relaxation arising from a continuous sum of exponential decays. *Phys. Rev. B* **2006**, *74*, 184430.

(14) Mzyk, A.; Sigaeva, A.; Schirhagl, R. Relaxometry with nitrogen vacancy (NV) centers in diamond. *Acc. Chem. Res.* **2022**, *55*, 3572–3580.

(15) Phillips, J. C. Stretched exponential relaxation in molecular and electronic glasses. *Rep. Prog. Phys.* **1996**, *59*, 1133.

(16) Stillinger, F. H. A topographic view of supercooled liquids and glass formation. *Science* **1995**, *267*, 1935–1939.

(17) Monceau, P. Electronic crystals: an experimental overview. *Adv. Phys.* **2012**, *61*, 325–581.

(18) Bodunov, E. N.; Antonov, Y. A.; Simões Gamboa, A. L. On the origin of stretched exponential (Kohlrausch) relaxation kinetics in the room temperature luminescence decay of colloidal quantum dots. *J. Chem. Phys.* **2017**, *146*, 114102.

(19) Vedelaar, T. A.; Hamoh, T. H.; Martinez, F. P. P.; Chipaux, M.; Schirhagl, R. Optimizing data processing for nanodiamond based relaxometry. *Adv. Quantum Technol.* **2023**, *2023*, 2300109.

(20) Pellet-Mary, C.; Perdriat, M.; Huillery, P.; Hétet, G. Relaxation processes in dipole-coupled nitrogen-vacancy centers in zero field: application in magnetometry. *Phys. Rev. Appl.* **2023**, *20*, 034050.

(21) Tetienne, J.-P.; Hingant, T.; Rondin, L.; Cavaillès, A.; Mayer, L.; Dantelle, G.; Gacoin, T.; Wrachtrup, J.; Roch, J.-F.; Jacques, V. Spin relaxometry of single nitrogen-vacancy defects in diamond nanocrystals for magnetic noise sensing. *Phys. Rev. B* **2013**, *87*, 235436.

(22) Myers, B. A.; Das, A.; Dartiailh, M. C.; Ohno, K.; Awschalom, D. D.; Bleszynski Jayich, A. C. Probing surface noise with depth-calibrated spins in diamond. *Phys. Rev. Lett.* **2014**, *113*, 027602.

(23) Sangtawesin, S. et al. Origins of Diamond Surface Noise Probed by Correlating Single-Spin Measurements with Surface Spectroscopy. *Physical Review X* **2019**, *9*, 1–17.
